# *ggtranscript*: an R package for the visualization and interpretation of transcript isoforms using *ggplot2*

**DOI:** 10.1093/bioinformatics/btac409

**Published:** 2022-06-25

**Authors:** Emil K Gustavsson, David Zhang, Regina H Reynolds, Sonia Garcia-Ruiz, Mina Ryten

**Affiliations:** Genetics and Genomic Medicine, Great Ormond Street Institute of Child Health, University College London, London WC1N 1EH, UK; Aligning Science Across Parkinson’s (ASAP) Collaborative Research Network, Chevy Chase, MD 20815, USA; Genetics and Genomic Medicine, Great Ormond Street Institute of Child Health, University College London, London WC1N 1EH, UK; Aligning Science Across Parkinson’s (ASAP) Collaborative Research Network, Chevy Chase, MD 20815, USA; Genetics and Genomic Medicine, Great Ormond Street Institute of Child Health, University College London, London WC1N 1EH, UK; Aligning Science Across Parkinson’s (ASAP) Collaborative Research Network, Chevy Chase, MD 20815, USA; Genetics and Genomic Medicine, Great Ormond Street Institute of Child Health, University College London, London WC1N 1EH, UK; Aligning Science Across Parkinson’s (ASAP) Collaborative Research Network, Chevy Chase, MD 20815, USA; NIHR Great Ormond Street Hospital Biomedical Research Centre, University College London, London WC1N 1EH, UK; Genetics and Genomic Medicine, Great Ormond Street Institute of Child Health, University College London, London WC1N 1EH, UK; Aligning Science Across Parkinson’s (ASAP) Collaborative Research Network, Chevy Chase, MD 20815, USA; NIHR Great Ormond Street Hospital Biomedical Research Centre, University College London, London WC1N 1EH, UK

## Abstract

**Motivation:**

The advent of long-read sequencing technologies has increased demand for the visualization and interpretation of transcripts. However, tools that perform such visualizations remain inflexible and lack the ability to easily identify differences between transcript structures. Here, we introduce *ggtranscript*, an R package that provides a fast and flexible method to visualize and compare transcripts. As a *ggplot2* extension, *ggtranscript* inherits the functionality and familiarity of *ggplot2* making it easy to use.

**Availability and implementation:**

*ggtranscript* is an R package available at https://github.com/dzhang32/ggtranscript (DOI: https://doi.org/10.5281/zenodo.6374061) via an open-source MIT licence. Further documentation is available at https://dzhang32.github.io/ggtranscript/.

## 1 Introduction

Alternative splicing is a crucial post-transcriptional step through which introns are excised from messenger RNA (mRNA) precursors, and exons are spliced together to form mature mRNA isoforms. In fact, ∼95% of human genes undergo alternative splicing resulting in various forms of mature mRNA (Wang *et al.*, 2008). This process is often regulated in a tissue-specific, disease-specific or developmental manner, resulting in multiple different transcripts being generated from the same gene.

It is well-recognized that it is challenging to identify full-length transcript structures from standard transcriptomic assays relying on short-read RNA-sequencing, as short-reads rarely span multiple splice junctions and therefore make it difficult to infer transcript structures (Conesa *et al.*, 2016). However, long-read sequencing platforms such as PacBio and Oxford Nanopore have transformed the field and enabled the discovery of new transcript isoforms that could not have been recognized by the assembly of short-reads. In addition, long reads facilitate better transcript quantifications and improve mapping of highly homologous sequences.

Current tools to visualize transcript structures are often inflexible, allowing users very limited control over the outputted plot aesthetics or lack the ability to compare transcript structures. For example, *UCSC genome browser* (Kent *et al.*, 2002), *IGV Browser* (Robinson *et al.*, 2011) and *Gviz* (Hahne and Ivanek, 2016) are genome-based tracks that allow for visualization of transcripts, but are not accessible programmatically. *IsoformSwitchAnalyzeR* (Vitting-Seerup *et al.*, 2019), *wiggleplotr* and *ggsashimi* (Li *et al.*, 2018) offers limited customization of plot aesthetics and comparisons of transcript structures. *SWAN* (Reese and Mortazavi, 2021) does offer some customizable transcript visualization functions, but has limited functionality to highlight differences and is within the python framework.

Here, we introduce the R package *ggtranscript* which makes it easy to both visualize and compare transcript structures using *ggplot2* (Wickham, 2016), a popular R-based framework for data visualization based upon an intuitive grammar system that permits flexibility via combination of independent components. As a *ggplot2* extension, *ggtranscript* inherits a vast amount of flexibility when determining the plot aesthetics, as well as interoperability with existing *ggplot2* geoms and *ggplot2* extensions. Furthermore, the input data for *ggtranscript* matches widely used formats in genetic and transcriptomic analyses.

## 2 Implementation

*ggtranscript* is an R package released that extends the incredibly popular tool *ggplot2* (RRID: SCR_014601 version: 3.3.5, https://cran.r-project.org/web/packages/ggplot2/index.html) for visualizing transcript structure and annotation.

As a *ggplot2* extension, the input data for *ggtranscript* are required to be a data.frame with columns specifying the start and end positions of each feature (e.g. exon or intron) as well as identifiers for the transcript(s) to be plotted. This data format is widely used across transcriptomic and genetic analyses and matches annotation and data structures such as the GTF/GFF3 files or GenomicRanges objects.

**Fig. 1.**
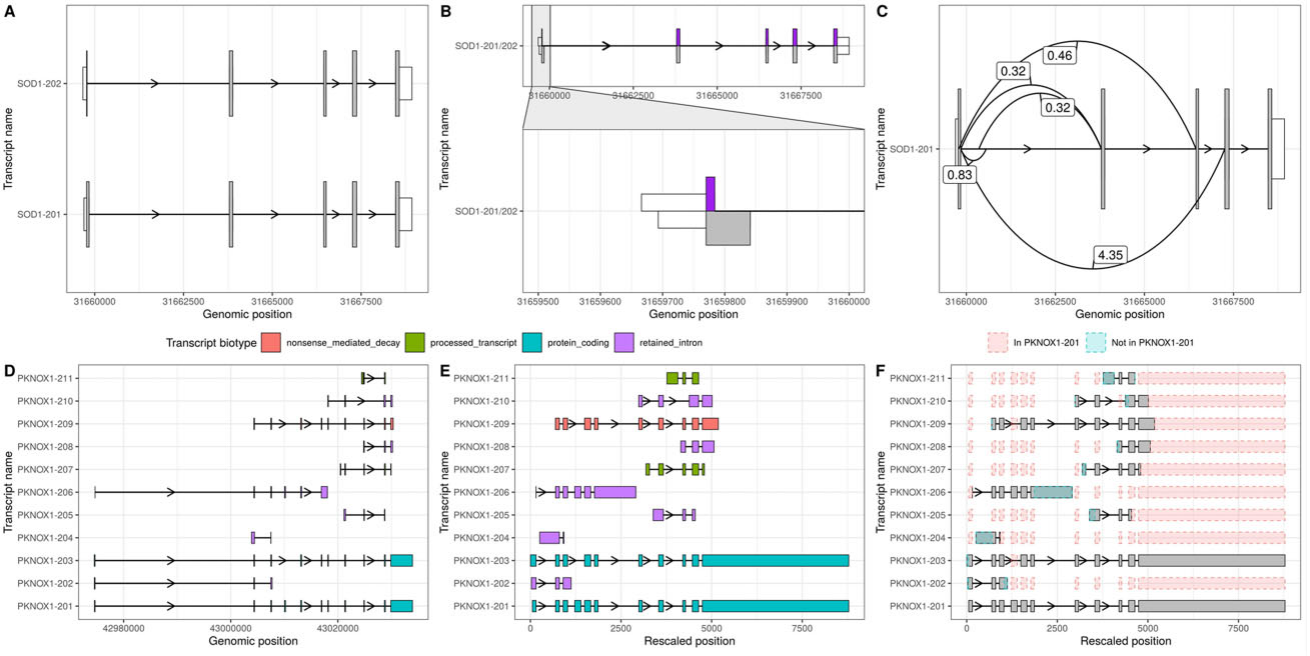
ggtranscript enables a fast and flexible method to visualize and compare transcript isoforms. ggtranscript is a ggplot2 extension that introduces five new geoms and a set of helper functions: (**A**) geom_range() and geom_intron() enable the plotting of exons and introns, the core components of transcript annotation. In addition, geom_range() has been used to visually distinguish coding regions from untranslated regions. (**B**) geom_half_range() enables users to plot only half of a range on the top or bottom of a transcript structure; one use case of which is to visualize the differences between two transcripts (SOD201 and SOD202). (**C**) geom_junction() enables the plotting of junction curves, which can be overlaid across transcript structures to annotate them with supporting short-read RNA-sequencing data. The number represent junction usage. (**D**) Longer, more complex transcripts, with small differences between exons of interest, can be more difficult to visualize. (**E**) For this reason, ggtranscript includes a helper function shorten_gaps() which shortens regions that do not overlap an exon to a fixed, user-inputted width. Transcripts in D and E are coloured by their transcript biotype. (**F**) In addition, the function to_diff() facilitates visualization of longer transcripts by highlighting differences in comparison to a reference transcript

To enable the visualization of the transcript structures, *ggtranscript* introduces five new geoms [geom_range(), geom_half_range(), geom_intron(), geom_junction() and geom_junction_label_repel()] and several helper functions designed to facilitate the visualization of transcript structure and annotation.

geom_range() and geom_intron() enable the plotting of exons and introns, the core components of transcript annotation (Fig. 1A). *ggtranscript* also provides the helper function to_intron(), which converts exon co-ordinates to the corresponding introns. Together, *ggtranscript* enables users to plot transcript structures with only exons as the required input and only a few lines of code. geom_range() is designed to be used for any range-based genomic annotation. For instance, when plotting protein-coding transcripts, geom_range() can be used to visually distinguish the coding regions from untranslated regions (Fig. 1A).

geom_half_range() takes advantage of the vertical symmetry of transcript annotation by plotting only half of a range on the top or bottom of a transcript structure; one use case of which is to visualize the differences between two transcripts more clearly (Fig. 1B). As a *ggplot2* extension, *ggtranscript* inherits the familiarity and functionality of *ggplot2*. For instance, by leveraging ggforce::facet_zoom() users can zoom in on regions of interest (Fig. 1B). geom_junction() enables the plotting of junction curves, which can be overlaid across transcript structures to annotate them with supporting short-read RNA-sequencing data (Fig. 1C). geom_junction_label_repel() adds a label to junction curves, which can often be useful to mark junctions with a metric of their usage such as read counts (Fig. 1C).

For longer, more complex transcripts, small differences between exons of interest can be more difficult to visualize (Fig. 1D). For this reason, *ggtranscript* includes a helper function shorten_gaps() which shortens regions that do not overlap an exon to a fixed, user-inputted width. Plotting of the rescaled exons and introns enables easier comparison between transcript structures when genes are long (Fig. 1E). In addition, the function to_diff() facilitates this by highlighting differences in comparison to a reference transcript (Fig. 1F).

Together, *ggtranscript* simplifies the process of visualizing and comparing transcript structures, facilitating the exploration, analyses and interpretation of long-read sequencing and transcriptomic data.

## 3 Conclusion

*ggtranscript* enables a fast and simplified way to visualize, explore and interpret transcript isoforms. It allows users to combine data from both long-read and short-read RNA-sequencing technologies, making systematic assessment of transcript support easier. Finally, by being a *ggplot2* extension it is highly flexible and can easily generate high-quality and publication-ready plots.
